# Pioneering AI Innovations to Transform Aging and Gerontological Care

**DOI:** 10.1093/geroni/igaf122.1467

**Published:** 2025-12-31

**Authors:** Walter Boot

## Abstract

The rapid advancement of artificial intelligence (AI) is reshaping society, offering unprecedented opportunities for innovation. In aging and gerontological care, AI holds transformative potential to enhance research, improve clinical decision-making, and support the health and well-being of older adults. This symposium highlights cutting-edge work applying AI to challenges in aging populations. Dr. Ian B. Kwok will introduce Clinical SmartReporter™, an AI-powered tool designed to enhance serious illness communication through real-time, patient-facing transcripts of family meetings. Yijung Kim will present an analysis of social determinants of health among homebound older adults, demonstrating how large language models like GPT-3.5 can support—but not fully automate—social needs identification. Dr. Amy M. Schuster will share survey findings showing that older adults’ attitudes toward AI significantly influence their likelihood of using it in healthcare, with implications for improving acceptance through targeted interventions. Taekyung Kim will discuss age-related stereotypes in GPT-4o-generated content, revealing lower perceived competence in descriptions of older adults and calling for strategies to reduce ageism in AI systems. Dr. Bo Xie will present a multi-agent large language model framework for streamlining systematic reviews of AI interventions for older adults, showing how hybrid human–AI collaboration can accelerate evidence synthesis. Collectively, these talks explore how AI can be thoughtfully integrated into aging research and care to promote equity, effectiveness, and inclusion. Technology and Aging Interest Group Sponsored Symposium

